# Novel homozygous frameshift variant in the *ATCAY* gene in an Iranian patient with Cayman cerebellar ataxia; expanding the neuroimaging and clinical features: a case report

**DOI:** 10.1186/s12920-023-01643-3

**Published:** 2023-09-26

**Authors:** Elham Salehi Siavashani, Mahmoud Reza Ashrafi, Homa Ghabeli, Morteza Heidari, Masoud Garshasbi

**Affiliations:** 1 PardisGene Co., Tehran, Iran; 2 Department of Stem Cells and Developmental Biology, Cell Science Research Center, Royan Institute for Stem Cell Biology and Technology, ACECR, Tehran, Iran; 3 Department of Pediatric Neurology, Pediatrics Center of Excellence, Children’s Medical Center, Tehran University of Medical Sciences, Tehran, Iran; 4 Pediatric Neurology Division, Myelin Disorders Clinic, Children’s Medical Center, Pediatrics Center of Excellence, Tehran University of Medical Sciences, Tehran, Iran; 5 Department of Medical Genetics, Faculty of Medical Sciences, Tarbiat Modares University, Tehran, Iran

**Keywords:** Cayman cerebellar ataxia, *ATCAY*, c.883_884del, p.(Lys295AspfsTer52)

## Abstract

**Background:**

Pathogenic variants in the *ATCAY* gene are associated with a rare autosomal recessive disorder called Cayman cerebellar ataxia. Affected individuals display psychomotor retardation, cerebellar dysfunction, nystagmus, intention tremor, ataxic gait and dysarthric in some cases.

**Case presentation:**

Whole exome sequencing was performed for a 21-month-old girl suffering from developmental delay specifically in motor and language aspects, hypotonia, nystagmus, pes planus and strabismus. The detected variant in the patient was confirmed by family segregation analysis by Sanger sequencing in both of her parents. Previously three homozygous variants in the *ATCAY* gene (missense, splice site and frameshift deletion) have been reported in patients with Cayman cerebellar ataxia. Here we report the fourth homozygous variant and the second homozygous frameshift deletion in this gene to be associated with autosomal recessive Cayman cerebellar ataxia.

**Conclusion:**

The identification of this novel homozygous frameshift deletion in the *ATCAY* gene expands our understanding of the genetic landscape underlying Cayman cerebellar ataxia. Furthermore, the occurrence of this variant in Iran, in addition to Pakistan, signifies the importance of considering genotypic and phenotypic factors beyond ethnicity when studying this disorder. These findings contribute to the ongoing efforts to unravel the molecular basis of Cayman cerebellar ataxia and improve diagnostic approaches and potential therapeutic interventions.

## Background

The Cayman cerebellar ataxia is an autosomal recessive neurologic disorder represented by birth-onset hypotonia, variable psychomotor retardation, and cerebellar dysfunction, including nystagmus, intention tremor, dysarthria, ataxic gait, and truncal ataxia in the patients [1, 2].

The disorder was initially identified in an isolated region on Grand Cayman Island and believed to be restricted to this area. First, Brown et al*.*, in 1986 and then Nystuen et al*.*, in 1996 reported 26 and 19 individuals, respectively with Cayman cerebellar ataxia on Grand Cayman Island. All patients had psychomotor retardation, prominent cerebellar dysfunction manifest by nystagmus, intention tremor, ataxic gait and dysarthric was present in some affected individuals. Life span was normal. Brain computed tomography (CT) scan, performed in 4 patients, showed marked cerebellar hypoplasia, with the vermis more involved than the hemispheres [1, 3]. In the previous case reports, the mutated gene and the variants related to Cayman ataxia cerebellar disorder were not detected. For the first time, Bomar et al*.*, investigated the *ATCAY* gene locus 19p13.3 as the associated gene with the disorder. They also detect two homozygous variants in the *ATCAY* gene including a missense p.(Ser301Arg) and a splice site variants (c.965 + 3G > T) in the affected individuals. The variants segregation analysis and the carrier status of 40 family members were confirmed [2]. Manzoor et al*.*, in 2018 reported a multigenerational highly consanguineous Pakistani family (RDHR-04) in which 6 people had severe gait ataxia with onset in infancy. All also had pes planus. Additional more variable features included strabismus, possible ocular apraxia, bibrachial dystonia, bradykinesia, and distal muscle atrophy primarily in the feet. Brain imaging in 1 patient showed marked cerebellar atrophy. There was suspicion of cognitive impairment. It was the first observation of the disorder in a place other than Grand Cayman Island and also the first frameshift deletion in *ATCAY* gene p.(Asp201AlafsTer20) [4]. In this article, we report the fourth homozygous variant and the second homozygous frameshift deletion in this gene p.(Lys295AspfsTer52) to be associated with autosomal recessive Cayman cerebellar ataxia.

The *ATCAY* gene encodes a neuron-restricted protein called caytaxin. Caytaxin has a CRAL-TRIO motif named after cellular retinal and the TRIO guanine exchange factor. This motif is common to proteins that bind to lipophilic molecules. Variants in such proteins cause a vitamin-E responsive ataxia. Three dimensional studies of the caytaxin showed its ligand is more polar than vitamin E [2]. Caytaxin contains 371 amino acids and is also called BNIP-H, a brain-specific member of the BNIP-2 family. BNIP-H interacts directly with kidney-type glutaminase (KGA) and both have broadly overlapping expression in the hippocampus and cerebellum. KGA converts glutamine to glutamate as an important source of neurotransmitter [5]. So, BNIP-H binds to KGA and regulates the synthesis of glutamate at synapses during neurotransmission. Variants in the *ATCAY* and loss of its function lead to glutamate excitotoxicity and deregulation of glutamatergic activation, causing Cayman [6].

## Case presentation

### Clinical and neuroimaging findings

The patient is a 21-month-old girl who was presented to Myelin Disorders Clinic at Tehran University of Medical Sciences due to developmental delay; particularly in motor, and language aspects. She was the sixth child of consanguineous parents and was born at 40 weeks of gestational age through an uneventful vaginal delivery with birth weight and head circumference of 3000 g (Z-score -0.5 SD) and 33 cm (Z-score -0.7 SD), respectively.

Her mother had a history of two abortions with unknown etiology. One of her sisters had behavioral problems and one of her brothers had stuttering speech. The patient didn’t achieve her normal developmental milestones completely until now. At this age, she has acceptable social interactions, obeys one-step commands and need to be supported significantly in order to sit, and has the ability to make sounds. Her maximum speech ability does not exceed sounds’ expression. One of the chief complaints reported by her mother was her feeding and swallowing problems.

**Fig. 1:**
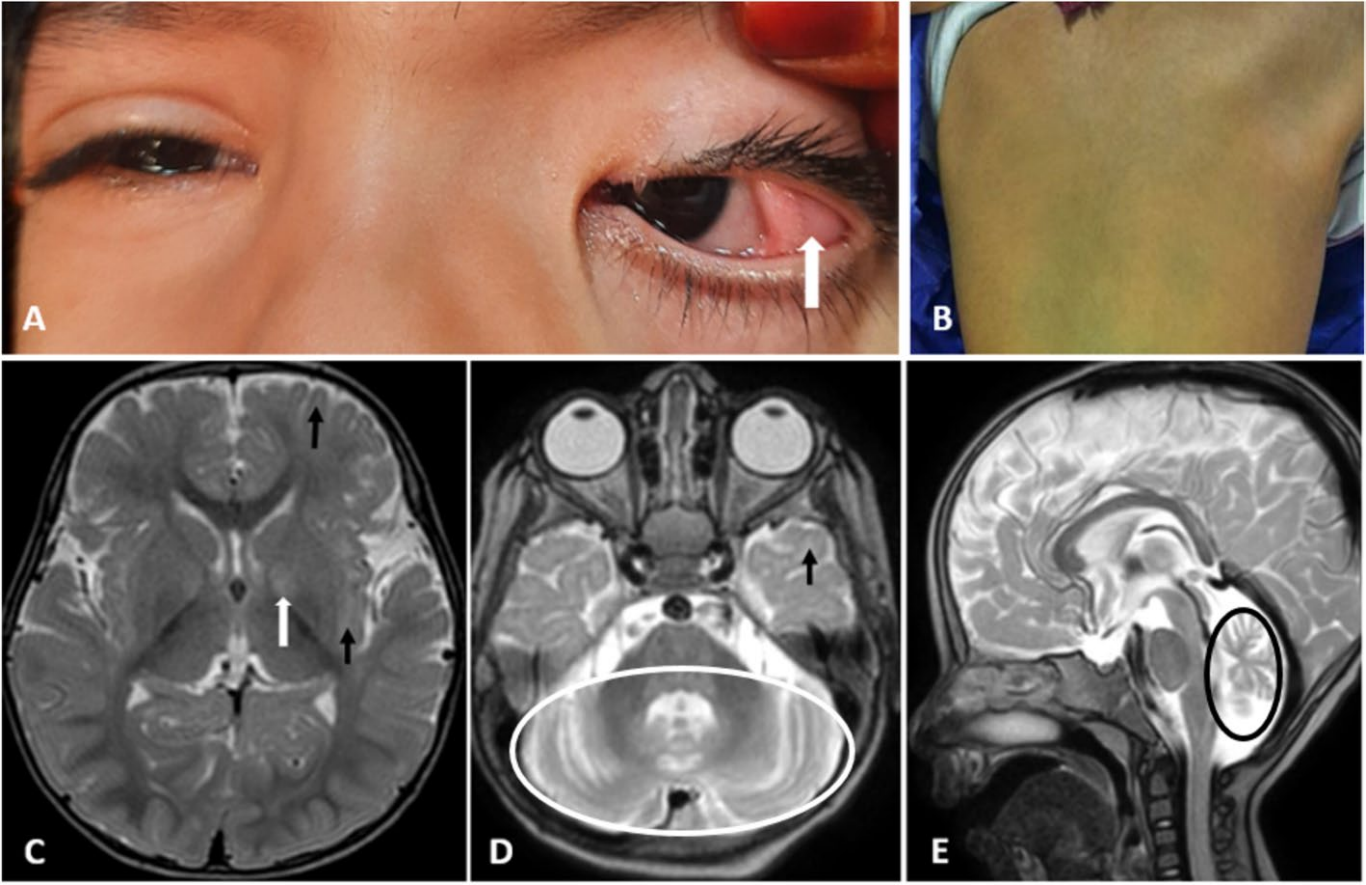
**A** a white subconjunctival lesion (lipodermoid), **B** diffuse melanosis on the back, **C-E** Brain MRI at the age of 16 months, axial and sagittal T2-Weighted Imaging sequence, illustrates delayed myelination; high in T2 WI is more marked in subcortical white matter in temporal and frontal lobes (**C**, **D**; black arrow), and hyperintensity of globus pallidus (**C**, white arrows). Cerebellar hemisphere (**D**, white circle) and cerebellar vermis (**E**, black circle) atrophy are additional findings

During the last clinical examination, she had a white subconjunctival lesion named lipodermoid on the lateral aspect of the left eye (Fig. 1A, white arrow), diffuse melanosis on the back (Fig. 1B), atrophy of buttock muscles in addition to strabismus, pendular nystagmus, truncal hypotonia, pes planus, and hyporeflexia with + 1 deep tendon reflexes (DTRs). Gross Motor Function Classification System (GMFCS) score was 3/5. Her weight was 7 kg (less than 3rd centile, Z-score -3.8 SD) and HC was 43.5 cm (Z-score -2.34 SD).

Basic metabolic tests including plasma lactate level, serum ammonia, Tandem Mass Spectroscopy (MS/MS), urine organic acid analysis and creatine kinase were all normal.

At 16 months of age, brain magnetic resonance imaging (MRI) revealed delayed myelination, along with the presence of hyperintensity in the globus pallidus, which had not been reported in previously documented cases. Additionally, there was observed atrophy of the cerebellar hemisphere and vermis (Fig. 1 C, D, E).

### Genetics findings

Whole exome sequencing and bioinformatics analysis was preformed as described previously [7]. Called variants were filtered considering minor allele frequency (MAF) > 0.01 based on the Gnome Aggregation Database (gnomAD v3) Consortium, and Iranome population databases [8, 9]. Benign annotated variants according to American College of Medical Genetics (ACMG) recommendations and clinical databases including Human Genome Mutation Database (HGMD) Professional and ClinVar (https://www.ncbi.nlm.nih.gov/clinvar/) were excluded from the analysis [10, 11]. The remaining variants were once again filtered based on the clinical information of the patient. Human Phenotype Ontology (HPO) database was used to extract associated genes with the patient’s phenotypes. The following HPO terms [12] were applied in the analysis: Generalized hypotonia (HP:0001290), Dysphasia (HP:0002357), Cerebellar atrophy (HP:0001272), Neurodevelopmental delay (HP:0012758), Abnormal corpus callosum morphology (HP:0001273), Brain imaging abnormality (HP:0410263), Fever (HP:0001945), Scanning speech (HP:0002168), Abnormality of the kidney (HP:0000077). The remaining variants were analyzed considering ACMG recommendations and the disease associated with each gene based on the Online Mendelian Inheritance in Men (OMIM) database. The candidate variant was checked in Integrative Genomics Viewer (IGV) software in order to see the depth of coverage and blat score of the reads showing the variant [13]. We ended up with a novel homozygous frameshift 2 nucleotides deletion in the *ATCAY* gene [NM_033064.5:c.883_884del, p.(Lys295AspfsTer52)] (Table 1).

**Table 1:** Detailed information of the identified variant in the *ATCAY* gene

Gene	Variant Coordinates	Zygosity	Allele Frequencies^a^	Type and Classification^b^	ACMG rules	Average coverage	%Target bp covered^c^
*ATCAY*	Chr19(hg38):g3913774 NM_033064.5:c.883_884del p.(Lys295AspfsTer52) Exon 9/13	Homozygous	gnomAD: NA ExAC: NA Iranome: NA	Frameshift deletion Pathogenic (Class 1)	PVS1 PM2 PP3	86.64	0X: 1.57 1X: 98.43 2X: 98.26 10X: 97.18 20X: 94.93 50X: 75.68

^a^Genome Aggregation Database (gnomAD) Genome version:3.0, Exome Aggregation Consortium (ExAC) version:1.0 and Iranome ^b^Variant classification is based on ACMG recommendations: Class 1: Pathogenic, Class 2: Likely pathogenic, Class 3: Variant of uncertain significance (VUS), Class 4: Likely benign, Class 5: Benign ^c^% Target bp Covered: the percentage of the covered target sequences based on the Agilent SureSelect Human All Exon V7 Kit. 0X: the percentage of the nucleotides with 0 coverage, 1X: the percentage of the nucleotides with 1 coverage and more, 2X: the percentage of the nucleotides with 2 coverage and more, 10X: the percentage of the nucleotides with 10 coverage and more, 20X: the percentage of the nucleotides with 20 coverage and more, 50X: the percentage of the nucleotides with 50 coverage and more

**Fig. 2:**
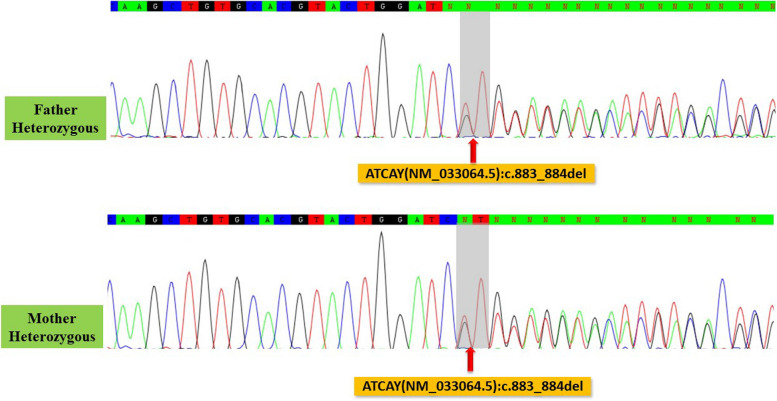
Sanger sequencing chromatographs shows heterozygous frameshift deletion in the parents

Genomic DNA of the proband’s parents were extracted. The genomic region covering the variant in the *ATCAY* gene was PCR-amplified using Forward primer: CATGGCTCTGTCTGGACCTA, and Reverse primer: GGCCCCTTAAACCTCTTTTG designed by online primer3Plus tool (https://www.bioinformatics.nl/cgi-bin/primer3plus/primer3plus.cgi). The PCR products were sequenced using a 3500 Applied Biosystems capillary electrophoresis machine. The sequencing chromatographs were analyzed using the CodonCode aligner software. The identified variant was confirmed by the segregation analysis in her parents (Fig. 2). It was classified as pathogenic based on the ACMG recommendations and considered as the cause of the disease in the proband.

## Discussion and conclusion

In 1986, Brown et al. were the first to report on Cayman, documenting 24 individuals with psychomotor retardation and cerebellar dysfunction on Grand Cayman Island. Following their work, Nystuen et al. reported 19 patients exhibiting similar phenotypes affected by Cayman cerebellar ataxia in 1996. Later in 2003, Bomar et al. investigated the *ATCAY* gene locus 19p13.3 as the associated gene locus with Cayman cerebellar ataxia, identifying two homozygous pathogenic variants: a missense variant [NM_033064.5:c.903C > G, p.(Ser301Arg)] and a splice site variant [NM_033064.5:c.965 + 3G > T] in the patients. Subsequently, in 2018, Manzoor et al. described six affected individuals from a highly consanguineous Pakistani family, displaying severe gait ataxia and cerebellar dysfunction. They identified a homozygous pathogenic frameshift variant with a 7-nucleotide deletion in the *ATCAY* gene [NM_033064.5:c.602_608del, p.(Asp201AlafsTer20)]. Here, we present a novel homozygous pathogenic frameshift variant involving a 2-nucleotide deletion in the *ATCAY* gene [NM_033064.5:c.883_884del, p.(Lys295AspfsTer52)] in a patient from Iran. This represents the second homozygous frameshift variant in the *ATCAY* gene and the second location, after Pakistan, outside of Grand Cayman Island (Table 2).

**Table 2:** Clinical features of the identified variants in the *ATCAY* gene

	Our variant (Frameshift deletion) (2 nucleotides)	Variant 1 (Missense)	Variant 2 (splice site)	Variant 3 (Frameshift deletion) (7 nucleotides)
Sex / age (year)	Female (1) / Less than 1 year	NA	NA	Male (6) / Less than 1 year
Onset	At birth	NA	NA	At birth
Origin	Iran	Grand Cayman Island	Grand Cayman Island	Pakistan
Psychomotor retardation	Y	Y	Y	Y
Nystagmus	Y	Y	Y	Y
Strabismus	Y	N	N	Y
Dysarthria	Y	Y	Y	Y
Hypotonia	Y	Y	Y	Y
Intention tremor	Y	Y	Y	Y
Truncal ataxia	Y	Y	Y	N
Ataxic gait	Y	Y	Y	Y
Pes planus	Y	Y	Y	Y
Extra neurological clinical features (subconjunctival lipodermoid, diffuse melanosis on the back, atrophy of buttock muscles, and failure to thrive)	Y	N	N	N
Cerebellar dysfunction / hypoplasia	Y	Y	Y	Y
Hyperintensity of globus pallidus	Y	N	N	N
Mutated gene	*ATCAY*	*ATCAY*	*ATCAY*	*ATCAY*
cDNA change	c.883_884del	c.903C > G	c.965 + 3G > T	c.602_608del
Amino acid change	p.(Lys295AspfsTer52)	p.(Ser301Arg)	-	p.(Asp201AlafsTer20)
Zygosity	Hom	Hom	Hom	Hom

*Abbreviations:* *Hom* Homozygous, *NA* Not Available

Besides common phenotypes observed in our patient and previously reported cases, she exhibited additional neurological features that warrant attention. These include the presence of a subconjunctival lipodermoid, diffuse melanosis on the back, atrophy of buttock muscles, failure to thrive, and a novel finding in the brain MRI indicating involvement of the globus pallidus, which has not been reported previously and may potentially represent a coincidental occurrence. Obtaining further reports on phenotypes from patients with similar gene mutations will provide valuable insights for a more accurate characterization of this disease and aid in making informed decisions. Mongolian spots or dermal melanosis are a type of birthmark that is commonly seen in infants and young children, especially in the Caucasian population, that is typically found on the lower back, buttocks, and sometimes other areas of the body, but persistent diffuse dermal melanosis is a diagnostic clue for some inborn errors of metabolism and few rare disorders. Although this finding may be an incidental sign in our patient we suggest that it may be an associated finding with Cayman cerebellar ataxia that was not reported till now. [14, 15]. This finding highlights the importance of further investigation and documentation of unique features associated with Cayman ataxia cerebellar. Understanding these additional neurological manifestations, such as the involvement of globus pallidus and ectopic melanocytes, can contribute to a more comprehensive understanding of the disorder and potentially aid in future diagnostic and therapeutic approaches.

## Data Availability

All data and materials applied in this study are stored in databases under the supervision of DeNA laboratory (http://dna-lab.ir/) and Pardis Gene Technology company (https://pardisgene.com/).

## Abbreviations

CT: Computed Tomography
CRAL-TRIO: Cellular Retinal and the TRIO guanine exchange factor
BNIP-H: Brain-specific member of the BNIP-2 family
KGA: Kidney-type Glutaminase
DTRs: Deep Tendon Reflexes
GMFCS: Gross Motor Function Classification System
MS/MS: Tandem Mass Spectroscopy
MRI: Magnetic Resonance Imaging
MAF: Minor Allele Frequency
gnomAD v3: Gnome Aggregation Database
ACMG: American College of Medical Genetics
HGMD: Human Genome Mutation Database
HPO: Human Phenotype Ontology
OMIM: Online Mendelian Inheritance in Men
IGV: Integrative Genomics Viewer
